# Hyperhomocysteinemia Causing Vascular Occlusion and Complicated by Heparin-Induced Thrombocytopenia and Gastrointestinal Bleed

**DOI:** 10.7759/cureus.72318

**Published:** 2024-10-24

**Authors:** Brooke A Finlayson, Megan Brooker, Marielle Roberts-McDonald, Penelope Mashburn

**Affiliations:** 1 Surgery, Ross University School of Medicine, Bridgetown, BRB; 2 General Surgery, Western Reserve Health Education, Northeast Ohio Medical University (NEOMED), Warren, USA

**Keywords:** atherosclerosis, cardiovascular disease, endothelial dysfunction, homocysteine, homocysteinemia, vascular occlusion

## Abstract

Homocysteine, an intermediate amino acid, is involved in methionine metabolism, which is crucial for various physiological pathways, including protein synthesis and DNA methylation. Elevated levels of homocysteine have been implicated as both a modifiable and unmodifiable risk factor in cardiovascular disease, influencing atherosclerotic disease formation and hypercoagulability. Mechanisms linking elevated homocysteine levels to vascular occlusion involve endothelial dysfunction, inflammation, and enhanced thrombotic potential. We present the case of a 54-year-old female with hyperhomocysteinemia-induced hypercoagulability causing acute limb ischemia, necessitating below-knee amputation.

## Introduction

Homocysteine (Hcy) is a sulfur amino acid produced during methionine metabolism, specifically as an intermediate of cysteine through the transsulfuration pathway and recycled back into methionine via the remethylation pathway [1,2]. The methylation reactions themselves are critical for protein synthesis and DNA methylation. Homocysteine is also seen as an intermediate in the S-adenosyl methionine (SAM) synthesis pathway, a molecule used in transmethylation, transsulfuration, and polyamine synthesis [1-3]. 

Cardiovascular disease (CVD) is the number one leading cause of death in the United States, with multiple modifiable and unmodifiable risk factors that change the prognosis and mortality rate in patients [4]. Homocysteine has been considered both a modifiable and unmodifiable risk factor of CVD since the early 1990s due to its pathogenesis, which includes genetic enzyme defects in metabolic biosynthesis as well as nutritional deficiencies of folate (vitamin B9), cobalamin (vitamin B12), and pyridoxine (vitamin B6) [1,2]. Only 50% of CVD cases explain the 'classic' risk factors such as diabetes, hypertension, obesity, and hyperlipidemia. Research has demonstrated that hyperhomocysteinemia may be a biomarker for atherosclerotic disease and hypercoagulability [1,2]. 

The case presented below describes a 54-year-old female who developed gradual right foot pain evolving over a month, conclusively diagnosed with a right-sided superficial femoral artery occlusion causing acute limb ischemia requiring below-knee amputation in the setting of hyperhomocysteinemia. This case highlights the importance of considering elevated levels of homocysteine as a potential contributor to the development of cardiovascular disease. 

## Case presentation

We present the case of a 54-year-old female with a previous medical history of seizures, hyperlipidemia, gastroesophageal reflux disease, and more than a 10-pack-year smoker who presented to the emergency department (ED) with approximately one month of right foot pain. Burning pain progressively worsened following onset and included claudication-like symptoms, swelling, decreased sensation, and decreased motor function. The patient was vitally stable at the time of presentation to the ED. Labs revealed mild leukocytosis, hypokalemia, mild anemia, and normal coagulation studies (Table 1). The femoral artery signal was the only signal detected by Doppler in the right lower extremity (RLE). In comparison, the left lower extremity had signals detected by Doppler in the femoral and popliteal arteries. 

**Table 1:** Relevant labs of the patient on admission to the hospital

Laboratory parameters	Patient values	Reference range
White blood cells	11.6 x10E3/uL	3.5-10.5 x10E3/uL
Neutrophils %	81.30%	34.0-71.0 %
Hemoglobin	11.5 gm/dL	12.0-15.5
Hematocrit	33%	34.9-44.5 %
Potassium	2.6 mmol/L	3.5-5.3 mmol/L
Prothrombin time (PT)	12.0 seconds	9.3-12.1 seconds
International normalized ratio (INR)	1.0	0.9-1.12
Activated partial thromboplast time (aPPT)	31.3 seconds	23.9-32.8 seconds
Platelets	126 x10E3/uL	150-450 x10E3/uL

**Figure 1:**
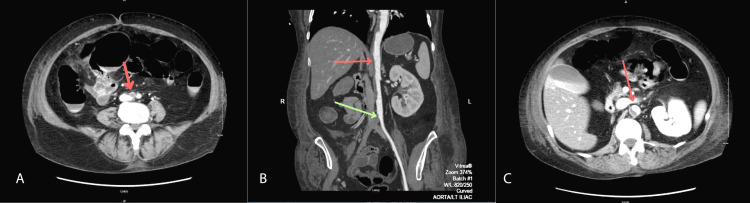
CT angiogram of aorta with run-off using intravenous contrast A: Axial view of right common iliac artery occlusion immediately distal to aortic bifurcation (red arrow); B: Coronal view of aortic plaque (red arrow) and common iliac artery occlusion (green arrow); C: Axial view of aortic plaque (red arrow)

Vascular surgery was consulted with a primary differential of acute on chronic RLE ischemia. A heparin drip was started, and a CT angiogram with run-off using intravenous contrast was ordered, revealing a right-sided superficial femoral artery occlusion in the proximal thigh (Figure 1A, Figure 1B). The left superficial femoral artery was occluded just beyond the origin of the artery. There was a large amount of soft plaque throughout the aorta on the posterior and right sides of the aortic wall (Figure 1B, Figure 1C). The patient was taken for emergent bilateral common femoral artery open thrombectomy and femoral-femoral bypass. 

The patient had regular neurovascular checks without any changes in the RLE's pulse status, motor, or sensation. She also began to have increasing pain from the RLE, and her creatine kinase and myoglobin increased (Table 2). Therefore, a fasciotomy was performed for the clinical suspicion of compartment syndrome. No improvements in her neurovascular examination were observed, and her RLE was deemed non-salvageable. The patient had an identifiable biphasic right popliteal pulse, so the decision was made to move forward with a below-knee amputation. 

**Table 2:** Myoglobin and creatine linase levels leading to fasciotomy

Laboratory parameters	Patient values	Reference range
Myoglobin hospital day 1	368 ng/mL	25-58 ng/mL
Myoglobin hospital day 2	7071 ng/mL	25-58 ng/mL
Myoglobin hospital day 3	3266 ng/mL	25-58 ng/mL
Creatine kinase hospital day 1	3906 ng/mL	38-234 ng/mL
Creatine kinase hospital day 2	11734 ng/mL	38-234 ng/mL
Creatine kinase hospital day 3	12790 ng/mL	38-234 ng/mL

Hematology was then consulted for a workup of a possible hypercoagulable disorder, revealing homocysteinemia at an elevated level of 143.0 µmol/L (reference range <13.0 µmol/L) (Table 3). The patient was started on folic acid and continued to receive a heparin drip throughout this time. She had a greater than 50% drop-off in her platelet count, so she was transitioned to argatroban for possible heparin-induced thrombocytopenia (HIT) (Table 4).

**Table 3:** Homocysteine level

Laboratory parameters	Patient values	Reference range
Homocysteine	143.0 umol/L	<13.0 umol/L

**Table 4:** Platelet levels from preoperative day one to postoperative day eight Platelet levels throughout the course of the patient's hospital stay led to the discontinuation of heparin and the initiation of argatroban following a significant drop in platelet levels. *Laboratory results were deemed unattainable due to platelet clumping, preventing accurate evaluation of platelet counts.

Laboratory parameters	Patient values	Reference range
Platelets preoperative day 1	113 x10E3/uL	150-450 x10E3/uL
Platelets postoperative day 1	Unattainable*	150-450 x10E3/uL
Platelets postoperative day 2	56 x10E3/uL	150-450 x10E3/uL
Platelets postoperative day 3	51 x10E3/uL	150-450 x10E3/uL
Platelets postoperative day 4	43 x10E3/uL	150-450 x10E3/uL
Platelets postoperative day 5	54 x10E3/uL	150-450 x10E3/uL
Platelets postoperative day 6	56 x10E3/uL	150-450 x10E3/uL
Platelets postoperative day 7	61 x10E3/uL	150-450 x10E3/uL
Platelets postoperative day 8	131 x10E3/uL	150-450 x10E3/uL

As the patient was recovering from her vascular surgery, she began to develop right-lower-quadrant abdominal pain. A CT of the abdomen and pelvis revealed free air compatible with bowel perforation. The patient was taken for an exploratory laparotomy, where a cecal perforation was found, and subsequently underwent an ileocecectomy. Three days after surgery, she began to pass bloody bowel movements. Her hemoglobin levels eventually decreased to 5.5 g/dL (normal range 12.0-15.5 g/dL) (Table 5). The patient received appropriate blood products, and anticoagulation was ceased. 

**Table 5:** Hemoglobin levels

Laboratory parameters	Patient values	Reference range
Hemoglobin hospital day 1	11.5 gm/dL	12.0-15.5 gm/dL
Hemoglobin hospital day 16	5.5 gm/dL	12.0-15.5 gm/dL
Hemoglobin hospital day 20	8.9 gm/dL	12.0-15.5 gm/dL

The patient then developed fingertip numbness and tingling near the end of her stay. This progressed to hand weakness, altered mental status, and blurry vision. She underwent a CT of the head and an MRI of the head and neck with no abnormalities found. A teleneurology consult was obtained with concern for a cerebrovascular accident. The patient continued to have bloody bowel movements and no improvement to her neurologic symptoms, prompting a transfer to a tertiary facility where she could be further worked up for her symptoms. 

## Discussion

Elevated serum or plasma levels of homocysteine are theorized to be secondary to impairments in metabolism, including cystathionine-synthase (CBS) deficiency or methylenetetrahydrofolate reductase (MTHFR) deficiency, certain disease states such as diabetes, or dietary deficiencies [1,2,5,6]. Elevated levels are considered a risk factor for the development of atherosclerosis and cardiovascular disease [1,2,6]. Hyperhomocysteinemia laboratory criteria vary by institution but are most commonly defined as having a level of 15.0 µmol/L or above circulating in the blood [1]. Serum levels between 5.0 and 15.0 µmol/L are considered normal, 16.0-30.0 µmol/L is moderate, 31.0-100.0 µmol/L is intermediate, and any levels exceeding 100.0 µmol/L are considered severe [1]. Described above is a case of vascular occlusion in the bilateral common femoral arteries secondary to severe hyperhomocysteinemia in a patient with a serum homocysteine level of 143.0 µmol/L. 

Mild to moderate elevations of plasma homocysteine have been reported to affect the coronary, cerebral, and retinal vasculature as a risk for atherosclerosis [7]. Moderate to severe homocysteine levels show evidence to be thrombogenic [1,6]. Elevated homocysteine levels correlate with an increased risk of venous thrombosis due to the promotion of platelet adhesion to endothelial cells [1,2]. One study on RBCs obtained from healthy adults involved exposure to homocysteine levels ranging from 8 to 800 μmol/L over 24 hours. The findings indicated a correlation between elevated homocysteine levels and increased phosphatidylserine exposure on RBCs, consequently leading to the formation of RBC procoagulant activity. This relationship was dose-dependent and statistically significant at the highest concentration of 800 μmol/L [1]. 

The exact mechanism of homocysteine and vascular occlusion is still unclear; however, several theories could be possible [1,8]. Homocysteine causes endothelial dysfunction or excessive smooth muscle cell proliferation (both seen in the atherosclerotic plaque formation pathway) by damaging the lining of blood vessels; this prevents the release of regulatory substances like nitric oxide, therefore leading to vasoconstriction and a decrease in blood flow [1,2,3,8]. Another theory reports that increased levels of homocysteine can increase the production of reactive oxygen species (ROS); this damages the endothelium of blood vessels and promotes inflammation, increasing the risk of plaque formation and rupture [1,2,3,8]. Homocysteine can also enhance the coagulation pathway by prompting platelet activation and clotting factor levels in the blood, ultimately leading to a prothrombotic state [1,6,8]. Another theory is related to elevated homocysteine levels impairing fibrinolysis; therefore, one with elevated levels of homocysteine is theorized to have a decreased ability to break down clots, increasing the risk of occlusion [8]. 

When the total homocysteine level is greater than 100 μmol/L, there appears to be an increased risk for an arterial or venous thrombotic event to occur, most commonly cerebral [1,6,9]. Diagnosing and treating homocysteinemia early in the disease course is important because up to 30% of homocysteine patients develop vascular events involving 33% cerebrovascular accidents, 25% pulmonary embolism, 15% peripheral venous thromboembolism, 11% peripheral arterial thromboembolism, and 4% myocardial infarction [9]. A study with 5,002 stroke patients and 4,945 controls demonstrated statistical significance (p < 0.01) with elevated homocysteine levels in patients with ischemic strokes [10]. This is theorized to be caused by the elevated homocysteine levels triggering necrosis, leading to endothelial dysfunction and ultimately interfering with the blood-brain barrier [10]. 

Those diagnosed with hyperhomocysteinemia earlier in life, particularly when the cause is secondary to CBS deficiency, can experience more severe and poorer outcomes. Previous studies have demonstrated that if a diagnosis is made by the age of 30, there is a mortality rate of 23% and a 30% chance of vascular events in hyperhomocysteinemia patients who do not undergo vitamin B6 treatment [5]. While treatment with B vitamins, including pyridoxine and folate, has been shown to decrease homocysteine, the impact on lowering the risk of vascular events is limited or negligible. This suggests that homocysteine may instead be a marker of disease but not a primary cause of vascular disease [5,6,8]. Another study concluded that while lowering homocysteine levels in CBS-deficient patients, it was not clear if lowering levels in patients with cardiovascular disease of unknown origin was beneficial [5,6]. 

## Conclusions

This study highlights the complex relationship between homocysteine levels and cardiovascular disease while illustrating the complex course of management when encountering multiple postoperative complications. Causes of elevated homocysteine include metabolic disorders, various disease states, or dietary deficiencies. While the mechanisms linking homocysteine to vascular pathology remain obscure, several theories suggest a role in endothelial damage, inflammation, and prothrombotic states. Treatment with B vitamins to reduce homocysteine serum levels has shown variable outcomes when applied to different causes of elevated homocysteine. Hyperhomocysteinemia as a differential diagnosis as a cause of cardiovascular disease should be included, particularly in a patient with an atypical presentation. 
